# Characterization of the complete chloroplast genome of *Eleutherococcus senticosus* (Araliaceae) as an herb in China

**DOI:** 10.1080/23802359.2020.1768929

**Published:** 2020-05-20

**Authors:** Zhuo Tian

**Affiliations:** College of Information Technology, Jilin Agricultural University, Changchun, China

**Keywords:** *Eleutherococcus senticosus*, chloroplast genome, genomics, phylogenetic analysis

## Abstract

*Eleutherococcus senticosus* is a highly valued woody herb medicinal plant belonging to the family Araliaceae, which is also the popular edible plant in China. In this paper, the chloroplast genome of *E. senticosus* was completed. The complete chloroplast genome of *E. senticosus* was 156,768 bp in length as a circle. It contained a large single-copy (LSC) region of 86,756 bp, a small single- copy (SSC) region of 18,154 bp and separated by two inverted repeat (IR) regions of 25,929 bp. The base compositions of chloroplast genome is uneven and the overall nucleotide composition is: A (30.7%), T (31.4%), C (19.3%) and G (18.6%), with a total G + C content (39.4%). It comprised 134 genes, including 89 protein-coding genes (PCGs), 37 transfer RNA (tRNA) genes and 8 ribosomal RNA (rRNA) genes. 8 PCG genes species, 7 tRNA genes species and 4 rRNA species were found duplicated in the IR regions. The phylogenetic analysis result shown that the chloroplast genome of *E. senticosus* is closest to *Eleutherococcus sessiliflorus* of the family Araliaceae in this study by the maximum-likelihood (ML) method. The complete chloroplast genome of *E. senticosus* can provide more genomics information for further research on the species in China.

*Eleutherococcus senticosus* is a highly valued woody medicinal plant belonging to the family Araliaceae, which is also the popular edible plant in China. *E. senticosus* is called Ci-Wu-Jia in Chinese that grows in the Russian Far East, Northeast China, Korea and Japan (Hwang et al. 2015). *E. senticosus* is popularly known as the Siberian ginseng because of its remarkable pharmacological effects, which can be used as a tonic and sedative and to treat rheumatism and diabetes (Umeyama et al. 1992). The main activities of *E. senticosus* are contains flavonoids, organic acids, phenols, triterpenoid saponins, lignans, coumarins, polysaccharides and other compounds (Wang et al. 2019). *E. senticosus* has been widely as an herb medicine used to treat neurasthenia, hypertension, immune regulation, cerebrovascular diseases and ischemic heart disease in China (Wang et al. 2019). Here, the complete chloroplast genome of *E. senticosus* was completed, which can be useful to study this species phylogenetic relationship and also can provide more genomics information for further research on the herb species in China.

The chloroplast DNA of *E. senticosus* was extracted from its fresh leaves which were sampled from the herb medicine market (Jilin, China, 127.30E; 42.44 N). The corresponding voucher herbarium specimen of *E. senticosus* was stored in Jilin Agricultural University Information Laboratory (No. JAUIL002) and the chloroplast DNA extracted using the Plant Tissues Genomic DNA Extraction Kit (TIANGEN, BJ, and CN). The chloroplast genome DNA was purified and sequenced, which was quality controlled and removed to the collected raw sequences. The chloroplast (cp) genome was assembled and annotated using the MitoZ (Meng et al. 2019). All the genes were analyzed using CPGAVAS (Liu et al. 2012) and combined with the online alignment tool Blastx and ORF Finder (NCBI). The tRNA genes were predicted using the online sites tRNA-scan (Lowe and Chan 2016). For the phylogenetic analysis, we selected and downloaded other 22 complete chloroplast genome sequences from the NCBI database. All the 23 chloroplast genome sequences were plants from NCBI. The annotated complete chloroplast genome sequence had been submitted to NCBI, the accession number is MK637765.2.

The complete chloroplast genome of *E. senticosus* sequence (MK637765.2) was 156,768 bp in length as a circle. The gene order and structure of the *E. senticosus* chloroplast genome is similar to the typical plant. It contained a large single-copy (LSC) region of 86,756 bp, a small single- copy (SSC) region of 18,154 bp and separated by two inverted repeat (IR) regions of 25,929 bp. The base compositions of chloroplast genome is uneven and the overall nucleotide composition is: A of 30.7%, T of 31.4%, C of 19.3% and G of 18.6% with a total G + C content of 39.4%. The chloroplast genome of *E. senticosus* comprised 134 genes, including 89 protein-coding genes (PCGs), 37 transfer RNA (tRNA) genes and 8 ribosomal RNA (rRNA) genes. 8 PCG genes species (*rpl23, rpl2, ycf2, ycf15, ndhB, rps7, rps12 and ycf1*), 7 tRNA genes species (*trnHis-GAU, trnLeu-CAA, trnVal-GAC, trnIle-GAU, trnAla-UGC, trnArg-ACG* and *trnAsn-GUU*) and 4 rRNA species (*rrn16, rrn23, rrn4.5* and *rrn5*) were found duplicated in the IR regions, respectively.

To study the phylogenetic relationships of *E. senticosus*, we constructed the maximum-likelihood (ML) phylogenetic tree using 23 plant species chloroplast genome sequences. Phylogenetic analysis was performed using RAxML software (Stamatakis 2014) with the best model, which used the number of bootstrap replicates with 2000. The ML phylogenetic tree was inferred with strong support and used the bootstrap values from 2000 replicates at all the nodes. The phylogenetic ML tree was drawn and edited using MEGA X (Kumar et al. 2018). As the phylogenetic analysis result (Figure 1), the chloroplast genome of *E. senticosus* is closest to *Eleutherococcus sessiliflorus* (KT153019) of the family Araliaceae in the phylogenetic relationship. This chloroplast genome of *E. senticosus* can be used to study this species genetic diversity and phylogenetic relationship that also can provide more genomics information for further research on the herb species in China.

**Figure 1.**
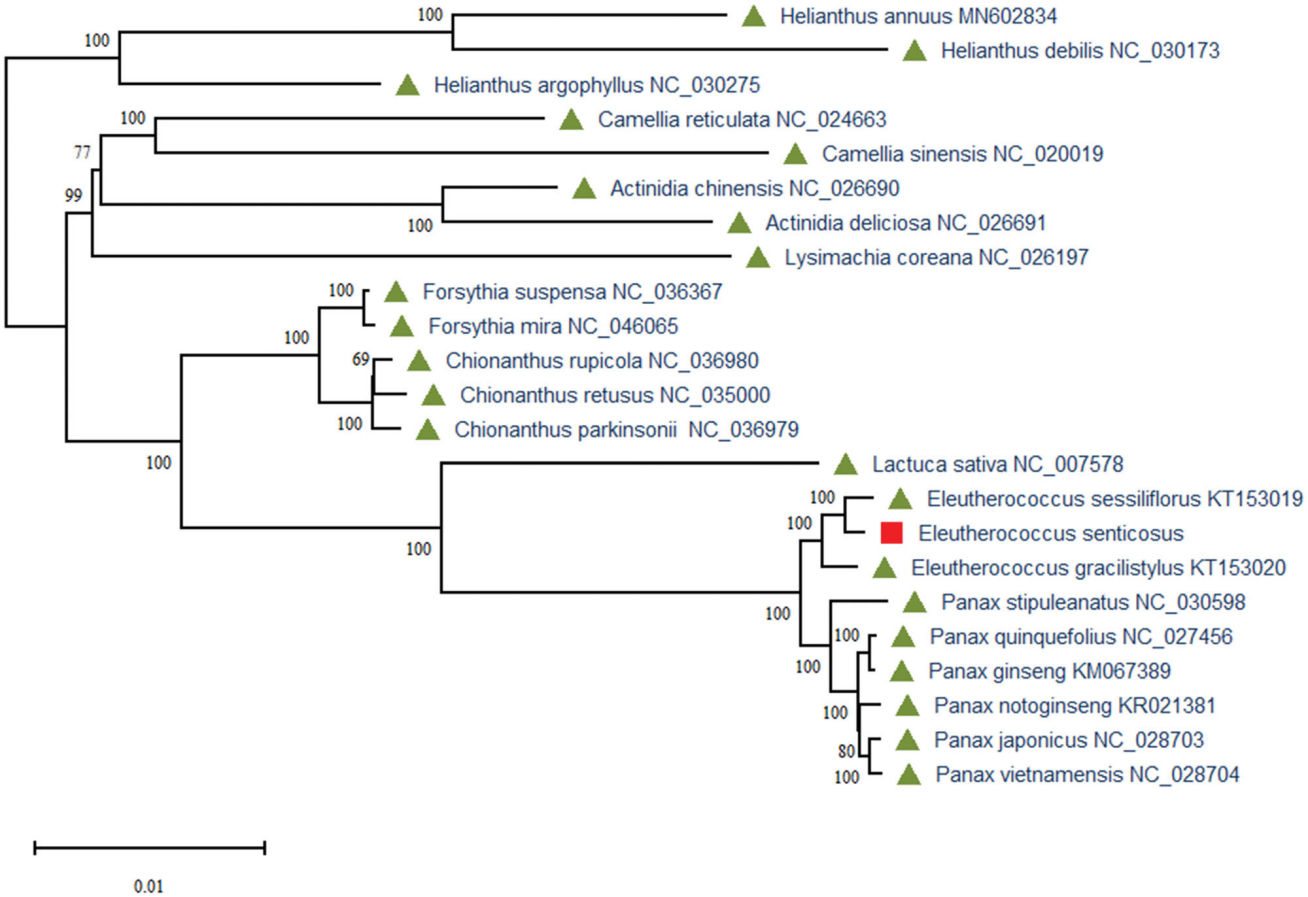
Maximum-likelihood (ML) phylogenetic tree based on 23 plant species chloroplast genome sequences using MEGA X. All the numbers at each node indicate the ML bootstrap values.

## Data Availability

The data that support the findings of this study are available from the corresponding author and upon reasonable request.

## Data openly available in a public repository that issues datasets with DOIs

The data that support the findings of this study are openly available in *Eleutherococcus senticosus* at http://doi.org/[doi], reference number is MK637765.2.
